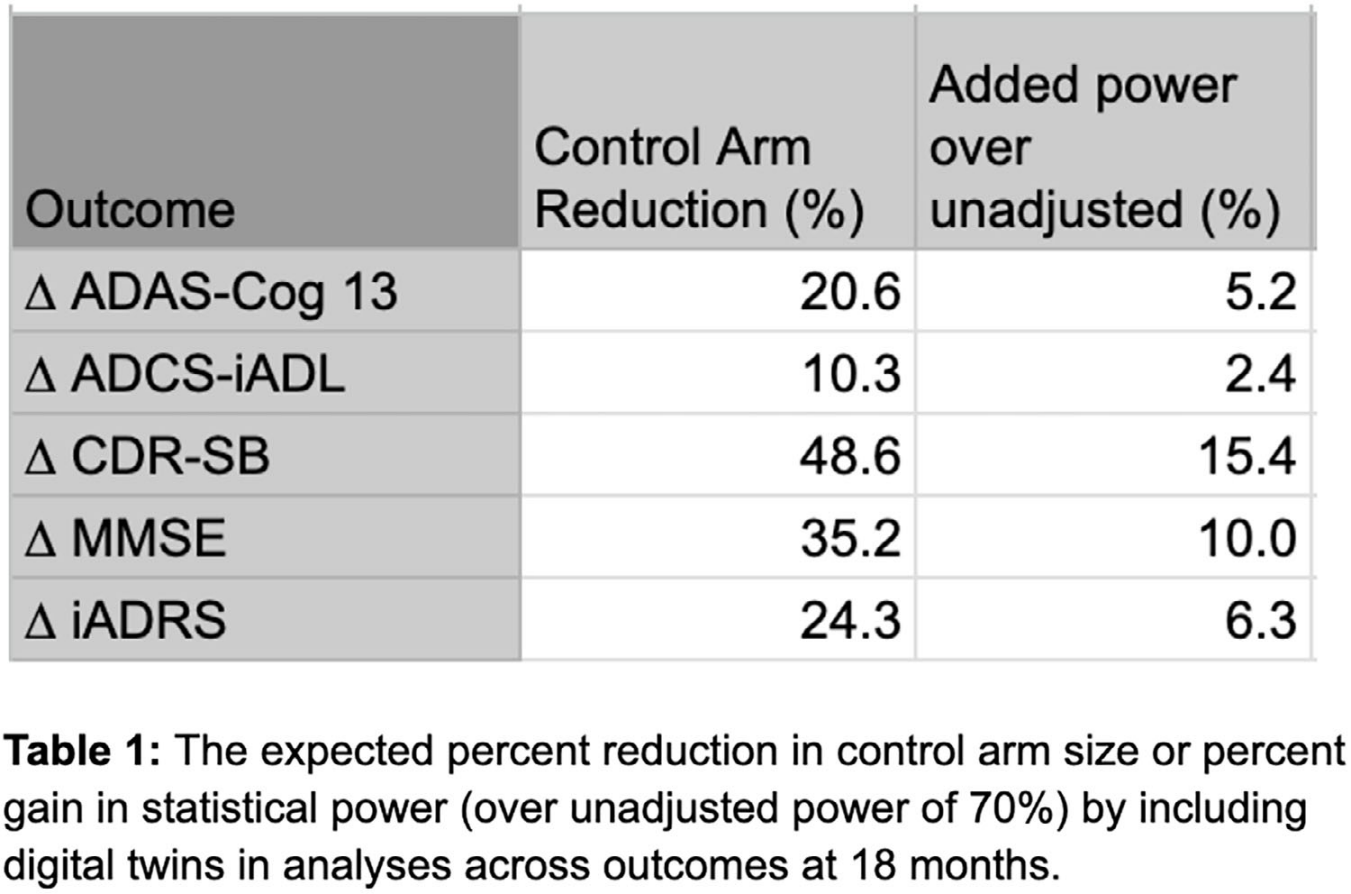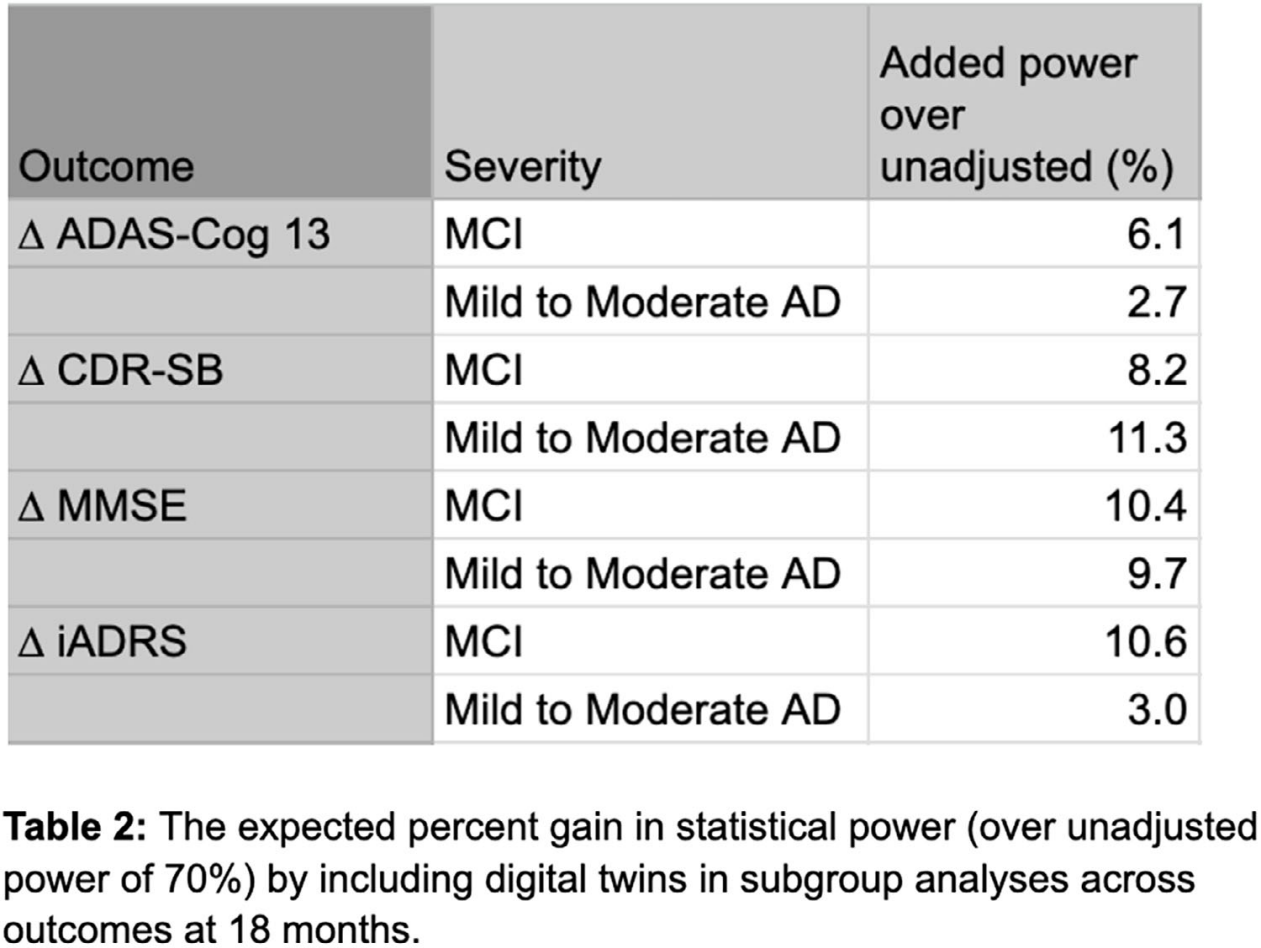# Leveraging digital twins for sample size reduction, powering secondary endpoints or subgroups in a simulated TRAILBLAZER‐ALZ 2 trial

**DOI:** 10.1002/alz70859_101285

**Published:** 2025-12-25

**Authors:** Coco Kusiak, Rachel Mak‐McCully, Carolyn Murray, Jonathan R. Walsh

**Affiliations:** ^1^ Unlearn.AI, San Francisco, CA USA

## Abstract

**Background:**

The TRAILBLAZER‐ALZ 2 (NCT04437511) trial demonstrated that donanemab reduces amyloid plaque burden and slows cognitive decline in a cohort with early symptomatic Alzheimer’s disease. The treatment effect was significant for the whole population on the primary endpoint. Despite testing in a large population, though, secondary endpoints and subgroup analyses of the primary were underpowered. Using a strong prognostic covariate, such as a forecasted outcome, in treatment effect analyses reduces the required sample size while maintaining statistical power or adds power without needing additional participants. Digital twins of participants are comprehensive, longitudinal forecasts of common clinical outcomes and biomarkers. The statistical method for prognostic score adjustment, PROCOVA, is qualified by the EMA and is concordant with FDA guidelines.

**Method:**

Here, we simulate the donanemab trial and quantify the sample size reduction or power gain that could have been attained had the analysis included digital twins. A synthetic dataset was constructed to reflect the TRAILBLAZER‐ALZ 2 cohort. First, participants that met the trial’s inclusion/exclusion criteria were selected from Unlearn’s harmonized data sources. Participants were then randomized 1:1 into experimental and control groups. Treatment effects simulating donanemab’s published impact were applied using a PCA‐based methodology. Digital twins were generated for each participant using Unlearn’s Alzheimer’s Disease Digital Twin Generator.

**Result:**

Due to the reduced variability in endpoints when using digital twins as covariates in statistical analyses, the TRAILBLAZER‐ALZ 2 trial could have enrolled up to 24% fewer control arm participants to draw the same conclusions about treatment efficacy as previously reported for the study’s primary outcome, the integrated Alzheimer Disease Rating Scale (iADRS) at 18 months. Alternatively, digital twins could be incorporated to increase power up to 15% for secondary endpoints. Potential sample size reductions and power gains are reported in Table 1. Similarly, subgroup analyses would gain power [Table 2]

**Conclusion:**

By including digital twins as a supercovariate in the analysis of a simulated donanemab trial, we demonstrate digital twins ability to reduce overall sample size or add power to secondaries and subgroups not factored into initial sample size calculations.